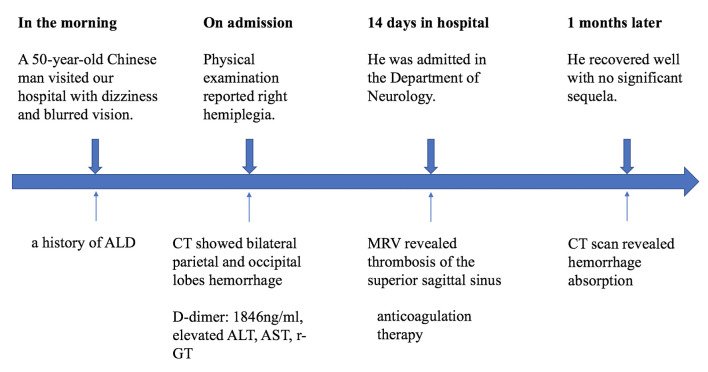# Cerebral venous thrombosis presented with symmetrical crescent-shaped intracranial hemorrhage in alcoholic liver disease: Case reports

**DOI:** 10.1097/MD.0000000000037441

**Published:** 2024-03-08

**Authors:** Lingjia Xu, Guoping Fu

**Affiliations:** a Department of Neurology, Shaoxing Second Hospital, The Second Affiliated Hospital of Shaoxing University Medical College, Shaoxing, Zhejiang, China.

**Keywords:** alcoholic liver disease, case reports, cerebral venous thrombosis, intracranial hemorrhage, magnetic resonance venography

## Abstract

**Rationale:**

Cerebral venous thrombosis (CVT) is a relatively uncommon but fatal disease. It can be caused by a variety of hereditary or acquired thrombotic diseases. Initial presentation with intracranial hemorrhage (ICH) in CVT is rare but can further complicate the therapeutic measures and prognosis. Cases of CVT presented with ICH in patients with alcoholic liver disease (ALD) have not been described in the literature, and it might be related with hemostatic abnormalities in ALD patients.

**Patient concerns:**

We report 2 cases of men admitted to our hospital who were diagnosed with CVT but initially presented with symmetrical crescent-shaped ICH; both of them were ALD patients.

**Diagnoses:**

Cerebral imaging revealed extended CVT in both cases. The first case was a 64-year-old man with ALD deteriorated with unconsciousness and convulsions; computed tomography showed symmetrical crescent-shaped ICH in the right temporal lobe, and magnetic resonance venography revealed CVT. Another 50-year-old man with ALD complained about dizziness and weakness of his right limbs; computed tomography revealed symmetrical crescent-shaped ICH in bilateral parietal and occipital lobes, and magnetic resonance venography revealed CVT.

**Interventions:**

The first patient was referred to the endovascular thrombectomy. Both of them were treated with anticoagulation treatment.

**Outcomes:**

Favorable outcomes were observed in both patients.

**Lessons:**

Symmetrical or multiple crescent-shaped ICH requires a high suspicion in the diagnosis of CVT; even with hemorrhage, it is still important to initiate anticoagulation therapy promptly. The crescent-shaped ICH might be a new sign for CVT, and further studies are needed in the underlying mechanisms of ALD and potential thrombophilia.

## 1. Introduction

Cerebral venous thrombosis (CVT) is a rare but serious cerebrovascular disease with high lethiferous and disable ratio.^[1]^ It is more common in young people, especially females. The etiology of CVT is complex; it can be caused by a variety of medical conditions and the majors are genetic or acquired coagulation dysfunction diseases, such as infection, tumor, brain trauma, pregnancy, oral contraceptives, thrombophilia, and so on.^[2]^ Initial presentation with intracerebral hemorrhage (ICH) in CVT patients is rare but can further complicate the treatment and prognosis.^[3]^

Alcoholic liver disease (ALD) is one of the most prevalent chronic liver diseases and the leading cause of cirrhosis after viral hepatitis or nonalcoholic fatty liver disease; ALD is typically diagnosed by a patient’s medical history, physical examination, imaging, laboratory tests, and liver biopsy is the gold standard for diagnosing ALD.^[4]^ Patients suffering from liver illness experience decreased absorption of vitamin K, which leads to the synthesis and depletion of various coagulation components, ultimately causing an imbalance between coagulation and anticoagulation function.^[5]^

Here we report 2 patients with CVT presented with symmetrical crescent-shaped ICH, and both have the underlying diseases of ALD, whether the thrombophilia mediated by ALD leads to CVT, is the focus of discussion and future research direction. Timely computed tomography (CT) or magnetic resonance venogram (MRV) examination has a pivotal position for better diagnosis and clinical treatment to improve the prognosis of patients.

## 2. Case reports

### 2.1. Case 1

**Figure 1.**
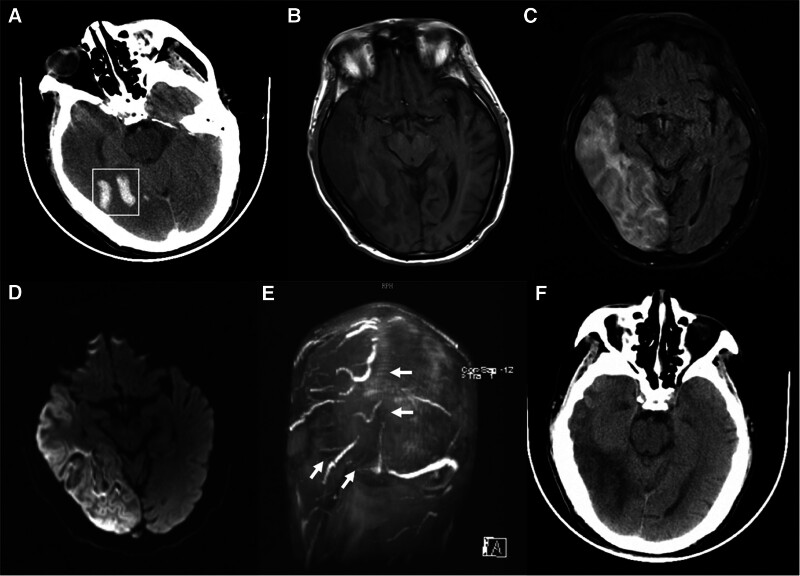
Case 1. (A and B) Non-contrast brain computed tomography (CT) scan and T1 hyperintensity of magnetic resonance imaging (MRI) suggest symmetrical crescent-shaped intracranial hemorrhage in the right temporal and occipital lobes (White box). (C) Fluid-attenuated inversion recovery (FLAIR) imaging shows juxtacortical edema surrounding the hemorrhage. (D) Diffusion weighted imaging (DWI) shows massive cerebral infarction throughout the right temporal and occipital lobes. (E) Magnetic resonance venography (MRV) imaging shows cerebral venous sinus thrombosis of the superior sagittal sinus, right transverse sinus, and sigmoid sinus (arrows). (F) CT examination obtained at 2-month follow-up.

A 64-year-old man who had a history of ALD (used to drink White wine 200 mL per day for about 40 years, equivalent to 166.4 g of pure alcohol per day), cirrhosis, and hypertension but had not received regular treatment, no family history of genetic diseases or traumatic history, went to our hospital’s emergency department for treatment after experiencing dizziness, nausea, vomiting, tinnitus, and drowsiness for 5 days without a fever or convulsions. The physical examination found the patient was conscious, well-oriented, had normal pulse with regular rhythm, and normal muscle strength, but the patient’s systolic and diastolic blood pressure measurements were 225 and 102 mm Hg, respectively. The real-time head CT found no obvious abnormalities. On laboratory examination, the D-dimer concentration increased (619 ng/mL, normal < 500 ng/mL), and the result of liver function test reported elevated alanine aminotransferase (120 U/L, normal < 40 U/L), aspartate aminotransferase (66 U/L, normal < 35 U/L), and r-glutamyl transferase (144 U/L, normal < 45 U/L). Other blood tests including blood routine test, thyroid and kidney function, rheumatism index, and tumor-related markers were all within the normal range. Urapidil hydrochloride injection and pantoprazole were applied emergently. Ten hours after admission, the patient suddenly lost consciousness with limb convulsions for about 5 minutes. He became delirious, failed to respond when calling and to cooperate in examination of muscle strength, and bilateral Babinski sign was negative. Urgent reexamination of the head CT showed symmetrical crescent-shaped ICH in the right temporal lobe (Fig. 1A). Since the hematoma shape was not in line with the general arterial hemorrhage, magnetic resonance imaging and MRV were performed and then revealed thrombosis of the superior sagittal sinus (SSS), right transverse sinus, and sigmoid sinus with corresponding hemorrhagic cerebral infarction (Fig. 1B–E). Emergent interventional thrombectomy was administrated. The patient was transferred to the intensive care unit after the surgery with continuous anticoagulation therapy and 1 week later, he was transferred to a rehabilitation institution with a modified Rankin Scale score of 3. He recovered well after the surgery with no significant sequela monitored in 2-month follow-up, his CT scan revealed hemorrhage absorption and an ancient infarction in the right temporal lobe (Fig. 1F and Figure S1, Supplemental Digital Content, http://links.lww.com/MD/L845 which indicates the timeline).

### 2.2. Case 2

**Figure 2.**
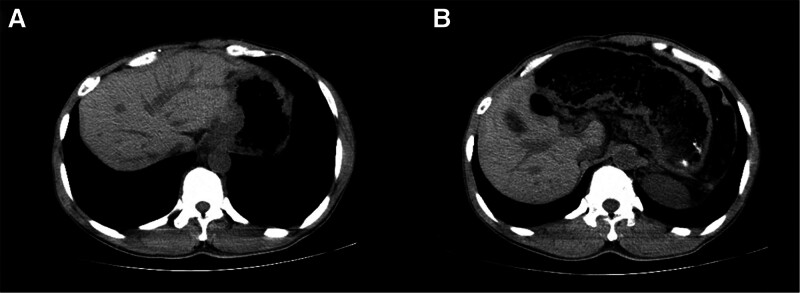
Abdominal CT revealed cirrhosis, liver lobar imbalance, and widened portal vein in Case 2. CT = computed tomography.

**Figure 3.**
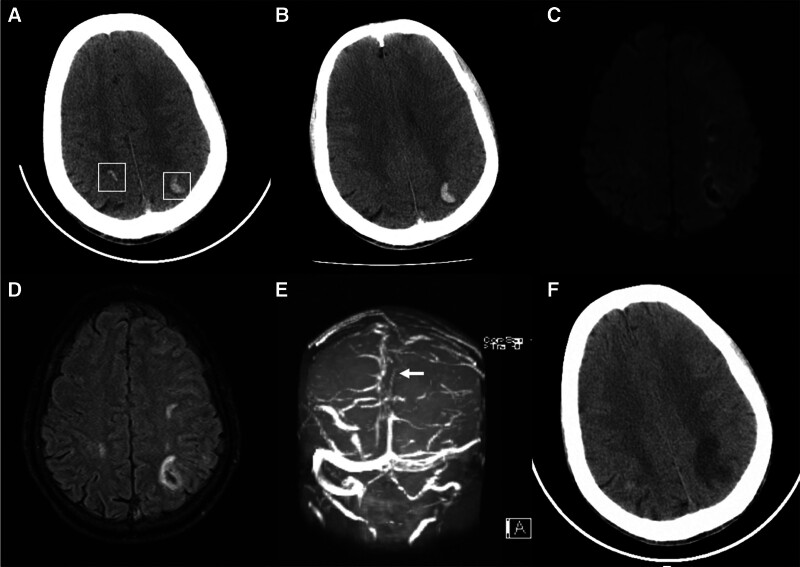
Case 2. (A and B) The brain CT scan shows symmetrical crescent-shaped intracranial hemorrhage in bilateral parietal and occipital lobes (White boxes). (C and D) DWI and FLAIR show corresponding multifocal cerebral infarction with hemorrhagic transformation. (E) MRV suggests superior sagittal sinus thrombosis (arrow). (F) CT examination obtained at 1-month follow-up. CT = computed tomography, DWI = diffusion weighted imaging, FLAIR = fluid-attenuated inversion recovery, MRV = magnetic resonance venography.

A 50-year-old man who was also an alcoholic (yellow rice wine ingestion for over 30 years, with a daily consumption of 1000 ml, equivalent to 128 g of pure alcohol per day) and diagnosed ALD presented to our emergency room with a sudden onset of dizziness accompanied by blurred vision, gradually progressive right limbs fatigue after getting up in the morning. He had no other diseases and drug use history, no family history, no genetic diseases, or traumatic history. During admission physical examination reported the patient was conscious, and had normal body temperature, respiratory rate, blood pressure, but right limbs hemiplegia (grade 1 of muscle strength), and positive right Babinski sign. His electrocardiography showed a pulse of 47 beats per minute indicating sinus bradycardia. The result of testing for the coagulation profile solely revealed a high concentration of D-dimer (1846 ng/mL). Residual blood test exclusively showed abnormal liver function with elevated alanine aminotransferase, aspartate aminotransferase, and r-glutamyl transferase (156, 78, and 256 U/L). Others including blood routine test, thyroid and kidney function, rheumatism index, and tumor-related markers reported no obvious abnormalities. His abdominal CT showed cirrhosis with liver lobar imbalance and widened portal vein (Fig. 2). Brain CT showed bilateral parietal and occipital lobes hemorrhage in consistent with the symmetrical crescent-shape (Fig. 3A and B). The brain magnetic resonance imaging found corresponding multifocal cerebral infarction with hemorrhagic transformation, MRV found SSS thrombosis (Fig. 3C–E). Anticoagulant therapy with dalteparin sodium, dehydration cranial pressure therapies with glycerol fructose and mannitol were applied, together with gastric mucosal protective agent, intravenous fluids, and rehabilitation at bedside. The patient’s condition improved after anticoagulation treatment in 1-month follow-up, and the CT showed hemorrhage absorption (Fig. 3F and Figure S2, Supplemental Digital Content, http://links.lww.com/MD/L846 which indicates the timeline).

## 3. Discussion

CVT is a relatively uncommon but fatal disease. Therefore, early diagnosis and prompt management are critical, and knowledge of its radiological features and potential pathology are essential to help it. ICH occurs in 30% to 40% of patients with CVT; CVT initially presented with ICH predicts a poorer prognosis and might be the leading cause of death in CVT patients,^[6]^ however, it remains elusive about the specific pathophysiology. On one hand, CVT impedes venous blood drainage, leading to increased venous pressure and subsequent brain tissue edema^[7]^; simultaneously, metabolites build up and interfere with neurovascular oxygenation, destroying the walls of capillaries and veins; this compromises the blood-brain barrier, causing blood to leak through and causing hemorrhagic transformation of cerebral venous infarction^[8]^; on the other hand, due to the absence of valve in cortical veins and smooth muscle in tunica media, the increased venous pressure during CVT leads to gradual dilation and even rupture of these veins, making blood extravasation to the brain parenchyma and eventually hemorrhage in multiple brain lobes.^[9]^

After viral hepatitis and nonalcoholic fatty liver disease, ALD is a kind of chronic liver disease and the primary cause of cirrhosis.^[10]^ A further potential cause of thromboembolism is liver dysfunction; studies have reported that various types of liver diseases are associated with an increased risk of developing thrombosis^[11]^; nevertheless, the mechanisms underlying how liver dysfunction leads to hypercoagulable states and how they translate into thrombotic events remain to be fully understood. In the issue of ALD, in addition to potentiating the growth of Gram-negative bacteria in the intestine and increasing intestinal permeability, alcohol consumption can damage liver cells by encouraging the accumulation of acetaldehyde and other reactive oxygen species in the liver, which may cause oxidative stress, metabolism disorders, and liver cell death. This ultimately raises the level of endotoxin in peripheral blood and causes inflammation, necrosis, and fibrosis in the liver.^[10,12]^ Proteases involved in the fibrinolytic system, anticoagulation factors, and coagulation factors are all synthesized in large quantities in the liver. Due to variable degrees of injury or disruption, liver cells’ capacity to produce coagulation and anticoagulation factors declines in ALD, leading to abnormalities of coagulation and anticoagulation mechanisms. In this context, patients with severe ALD may have both a higher risk of developing hemorrhage and thrombosis.^[13]^

CVT with concomitant ALD has not been reported yet; in the present report, both patients had ALD and a high D-dimer concentration without other coagulation indicators of abnormalities were detected. Whether the CVT represents a major threat of hemostatic abnormalities among ALD patients requires further exploration with more cases. Common cerebral hematomas are often single, round, and oval-shaped, in line with the distribution of cerebral arterial blood vessels, while ICH with diverse forms like the shape of cashew nuts, carob is often detected in CVT with hemorrhagic transformation.^[7,14]^ In our cases, the crescent-shaped juxtacortical hemorrhages may be related to the orientation of the subcortical vein along the arcuate fiber. The superficial and deep systems of the brain’s venous drainage merge into the subcortical veins and SSS in the cortex,^[15]^ and when the SSS is blocked by thrombus, the pressure of the subcortical veins increases and it is easy to rupture and lead to juxtacortical hemorrhages. Clinically, the symmetrical crescent-shaped ICH in the aforementioned images, whether or not it is in the same hemisphere, requires a high degree of suspicion when diagnosing CVT, and it is highly recommended to perform MRV examination to confirm the diagnosis earlier and improve the prognosis. Moreover, additional research is required to address the possibility of thrombophilia and ALD.

## 4. Conclusions

The symmetrical or multifocal crescent-shaped ICH in the aforementioned images raises serious doubts about the diagnosis of CVT, and it is highly recommended to perform MRV examination to confirm the diagnosis earlier and improve the prognosis. Prompt anticoagulation is crucial and advantageous for patients.

## Author contributions

**Conceptualization:** Lingjia Xu.

**Data curation:** Lingjia Xu.

**Investigation:** Lingjia Xu, Guoping Fu.

**Supervision:** Guoping Fu.

**Validation:** Guoping Fu.

**Writing – original draft:** Lingjia Xu.

**Writing – review & editing:** Lingjia Xu, Guoping Fu.

## Supplementary Material

**Figure:**
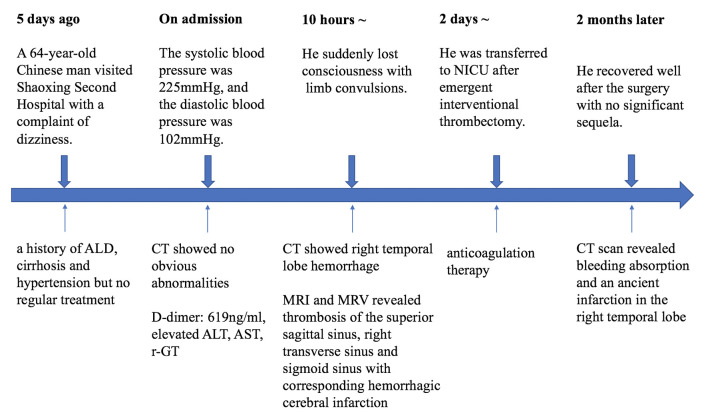


**Figure:**